# The DDIT4L-TOM40-ATP5A pathway suppresses glioblastoma oncogenesis

**DOI:** 10.1016/j.gendis.2026.102121

**Published:** 2026-03-04

**Authors:** Pu You, Zeng-Xin Qi, Hao Luo, Bing Cai, Xu-Ye Hu, Jing Pan, Pan-Pan Gan, Xin-Ying Zeng, Ri-Jing Liao, Qisheng Tang, Ye Yu, Zhen-Ning Zhang, Bojie Yang, Liang Chen, Xu Zhang, Kai-Cheng Li, Ying Mao

**Affiliations:** a Department of Neurosurgery, Huashan Hospital, Shanghai Medical College, State Key Laboratory of Medical Neurobiology, MOE Frontiers Center for Brain Science and Institutes of Brain Science, Fudan University, Shanghai 200040, China; b Institute of Brain-Intelligence Science and Technology, Zhangjiang Laboratory, Shanghai 201210, China; c Shanghai Clinical Research Center, Chinese Academy of Sciences/XuHui Central Hospital, Shanghai 200020, China; d School of Life Science and Technology, ShanghaiTech University, Shanghai 201210, China; e School of Traditional Chinese Pharmacy, China Pharmaceutical University, Nanjing, Jiangsu 210009, China; f School of Life Sciences and Technology, Tongji University, Shanghia 200092, China; g Tianqiao and Chrissy Chen Institute Clinical Translational Research Center, Shanghai 200040, China; h Shanghai QuietD Biotechnology Co., Ltd., Shanghai 201210, China

Glioblastoma (GBM) treatment resistance is closely linked to mitochondrial dysfunction,^1^ yet the inherent regulators coupling mitochondrial dynamics to apoptosis remain unclear. Our multi-modal investigation identified DNA damage-inducible transcript 4-like (DDIT4L) as a critical mitochondrial suppressor in GBM pathogenesis. Clinical analyses demonstrate that elevated DDIT4L expression correlated with prolonged patient survival. Mechanistically, we identified a unique import pathway through which DDIT4L was transported into mitochondria via single TOM40. Within mitochondria, DDIT4L interacted with the subunit of ATP synthase, thereby inhibiting mitochondrial function and inducing tumor cell apoptosis. The therapeutic relevance of this pathway was further validated by a synthetic mitochondrial-targeting peptide (DDIT4L^V125–P132^), which significantly suppressed tumor growth in patient-derived xenograft (PDX) models.

Differentially expressed genes were analyzed among different subtypes of gliomas. We identified 53 up-regulated genes by performing comparative analysis (Fig. S1A; Table S1). *DDIT4L* was one of the genes showing the most significant up-regulation in the IDH-wild-type and mesenchymal type of GBM (Fig. S1B–S1D), while its homolog *DDIT4* was highly expressed in lower grade glioma (LGG) (Fig. S1E). Given that DDIT4L exhibited robust induction under hypoxic conditions,^2^ a characteristic directly relevant to GBM biology, we selected it as our focus gene. We found that DDIT4L expression was lower in gliomas with better prognosis compared to adjacent tissues, but slightly higher in GBM (Fig. S1F). Analysis of the Chinese Glioma Genome Atlas (CGGA) and The Cancer Genome Atlas (TCGA) databases indicated that in patients with low-grade glioma, high DDIT4L expression was associated with a shorter median survival time, whereas in grade IV glioma, survival did not differ significantly across DDIT4L expression levels (Fig. S1G and S1H). In line with the bioinformatic analysis, immunohistochemical staining showed that DDIT4L expression was higher in GBM than in LGG (Fig. 1A). In GBM tissue, DDIT4L partially co-localized with GFAP or Oligo2 (Fig. 1B). DDIT4L was also detected in Nestin-, CD133-and SOX2-positive glioma-initiating cells (Fig. 1C; Fig. S2A and S2B). Taken together, DDIT4L was expressed at a much higher level in GBM than in other glioma subtypes.

**Figure 1:**
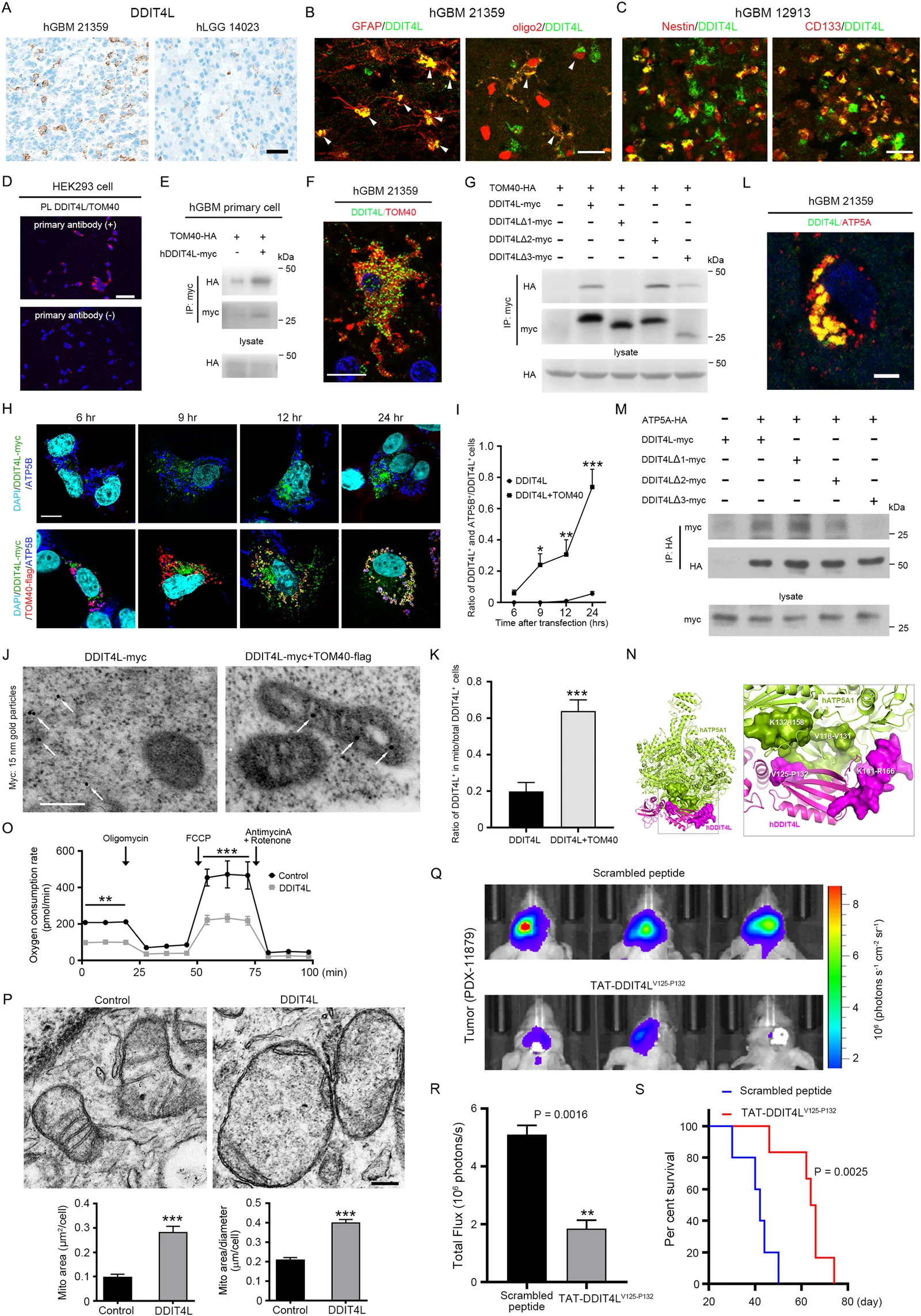
DDIT4L suppresses glioblastoma (GBM) oncogenesis. **(A)** Immunohistochemical staining of DDIT4L in GBM and lower grade glioma (LGG). Scale bar = 100 μm. **(B)** Double-immunostaining of DDIT4L and GFAP, DDIT4L and Oligo2 in GBM tissues. Scale bar = 50 μm. **(C)** Double-immunostaining of DDIT4L and Nestin, or DDIT4L and CD133 in GBM tissues. Scale bar = 100 μm. **(D)** Proximity ligation assay assay shows that DDIT4L binds to TOM40 in HEK293 cells. Scale bar = 20 μm. **(E)** Co-immunoprecipitation (Co-IP) in GBM primary cells (hGBM11879) co-expressing TOM40-HA and DDIT4L-myc: TOM40-HA is detected in the proteins precipitated with anti-myc antibody (*n* = 3). **(F)** Double immunostaining shows that DDIT4L is colocalized with TOM40 in GBM tissues. Scale bar = 20 μm. **(G)** Co-IP in HEK293 cells expressing TOM40-HA with DDIT4L-myc or DDIT4LΔ2-myc (Δ2: deleting region of V125–P132) or DDIT4LΔ3-myc (Δ3: deleting region of K141–R166). HA signal is detected in proteins precipitated with anti-myc antibody, and decreased in the cell co-expressing TOM40-HA and DDIT4LΔ1-myc (Δ1: deleting region of S96–C106) (*n* = 3). **(H)** DDIT4L overexpression alone did not localize to mitochondria after 24 h. When co-expressed withTOM40, DDIT4L mainly localized in TOM40-positive granule at 12 h, and the ATP5B positive inner membrane of mitochondria disappeared. Scale bar = 10 μm. **(I)** Quantification shows the ratio of DDIT4L positive and ATP5B positive cells/DDIT4L positive cells. **(J)** Electron microscopy showed that transfected DDIT4L in the HEK293 cells mainly dispersed in cytoplasm. While after co-transfection of DDIT4L and TOM40, DDIT4L mainly appeared in mitochondria, accompanied by mitochondrial swelling and cristae reduction. Scale bar = 200 nm. **(K)** Quantification shows the ratio of DDIT4L immunogold granule in mitochondria/total DDIT4L immunogold granule in cell body. **(L)** Double immunostaining shows that DDIT4L is co-localized with ATP5A in GBM tissues. Scale bar = 20 μm. **(M)** Co-IP in HEK293T cells expressing ATP5A-HA with DDIT4L-myc or DDIT4LΔ1-myc (Δ1: deleting region of S96–C106), myc signal is detected in proteins precipitated with anti-HA antibody. The Co-IP signal of myc is decreased in the cell co-expressing ATP5A-HA and DDIT4LΔ2-myc (Δ2: deleting region of V125–P132) or DDIT4LΔ3-myc (Δ3: deleting region of K141–R166) (*n* = 3). **(N)** The selected DDIT4L (purple)/ATP synthase (green) model is derived from clustering analysis of 2000 possible binding poses based on the ZDOCK scoring, and following optimization using molecular dynamics simulations. Possible motifs implicated in interactions between DDIT4L and ATP synthase are highlighted using purple and green surface modes, respectively. **(O)** Seahorse Mito Stress Kit assay showed that oxygen consumption rates (OCRs) on tumor cells transfected with the DDIT4L plasmid decreased when compared with that transfected with control plasmid (*n* = 3). **(P)** Representative EM images of the GBM cells transfected with the plasmid expressing DDIT4L show the mitochondrion fusion and reduction of mitochondrion crista after expressing DDIT4L. Scale bar = 200 nm. Quantification shows that the expressing of DDIT4L increases the mitochondria area and the ratio of area/diameter. **(Q)** Bioluminescence imaging of GBM patient derived tumor expressing the luciferase after treatment with TAT-DDIT4L^V125–P132^ or scrambled peptide show that TAT-DDIT4L^V125–P132^ suppress tumor growth. Representative images are captured from animals on day 30 after minipumps implanted. **(R)** Total Flux of photon emission is decreased by TAT-DDIT4L^V125–P132^ (*n* = 6 per group). **(S)** Kaplan–Meier survival analysis of mice with GBM xenografts shows that TAT-DDIT4L^V125–P132^ extends lifespan dramatically (*n* = 6 per group).

Because hypoxia is a feature of GBM and hypoxia up-regulates *DDIT4L* expression,^2^ DDIT4L protein levels in GBM were higher than in LGG, and increased gradually in primary GBM cells after hypoxic culture (Fig. S3A and S3B). In GBM tissues, DDIT4L primarily co-localized with HIF1α but not with CD31 (Fig. S3C). Flow cytometry analysis revealed that 36.3% of primary GBM cells were both DDIT4L-positive and HIF1α-positive, while only 5.01% were both DDIT4L- and CD31-positive (Fig. S3D). DDIT4L overexpression inhibited the proliferation of U87MG cells, while RNAi targeting *DDIT4L* increased cell proliferation (Fig. S3E and S3F). Moreover, DDIT4L overexpression significantly impaired the tumor sphere formation of primary GBM cells (Fig. S3G and S3H). These findings indicate that DDIT4L mainly localizes in hypoxia-sensitive GBM cells and suppresses GBM tumor growth.

To identify novel targets of DDIT4L in tumor cells, we transfected HEK293 cells with DDIT4L and immunoprecipitated the cell lysate with anti-DDIT4L antibody. Two bands with molecular weights of ∼40 and ∼50 kDa were coimmunoprecipitated (Figs. S4A and S7A). The mass spectrometry analysis represented 28% and 37% coverage of the TOM40 and ATP5A protein sequences, respectively. Co-immunoprecipitation, immunostaining assays and proximity ligation assays further confirmed that DDIT4L specifically interacted with TOM40,^3^ but not TOM20, TOM22 or TOM70 in GBM tissue or HEK293 cells (Fig. S4B and S4C; Fig. 1D–F). Then, we investigated the possible docking site of DDIT4L on TOM40 using a combination of homology modeling and *in-silico* docking. In this model, we identified three DDIT4L motifs as possible binding sites for TOM40 (Fig. S4D). The structural analysis showed that the S96–C106 domain of DDIT4L was a functional domain binding to TOM40 (Fig. S4E). Consistently, co-immunoprecipitation showed that the S96–C106 motif, but not the V125–P132 and K141–R166 motifs, in DDIT4L bound to TOM40 (Fig. 1G).

Next, we examined the subcellular distribution of DDIT4L. Immunostaining results showed that DDIT4L did not co-localize with mitochondria in cells transfected with DDIT4L (Fig. S5A). Surprisingly, when cells were co-transfected with DDIT4L and TOM40, DDIT4L mainly co-localized with mitochondria (Fig. S5B and S5C). Similarly, in COS7 cells transfected with DDIT4L, DDIT4L did not localize to the inner mitochondrial membrane (labeled with ATP5B) 24 h after transfection. When DDIT4L was co-transfected with TOM40, it mainly localized in TOM40-positive granules, and became detectable in the ATP5B-positive inner mitochondrial membrane (Fig. 1H and I). Moreover, in cells transfected with DDIT4L alone, it mainly co-localized with the lysosome marker LAMP1, whereas in cells co-transfected with DDIT4L and TOM40, it predominantly co-localized with MitoTracker (Fig. S6A and S6B).

Immunostaining showed that DDIT4L was co-expressed with ATP5A in GBM cells (Fig. 1L). Furthermore, co-immunoprecipitation assay showed that both ATP5A and ATP5B interacted with DDIT4L in GBM primary cells (Fig. S7B). Both GST pull-down and purified protein interaction experiment indicated that ATP5A is a direct target of DDIT4L (Fig. S7C and S7D). We next investigated the key motifs involved in the DDIT4L-mediated modulation of ATP synthase activation. We first identified a possible docking site of DDIT4L in the ATP synthase by homology modeling and *in-silico* docking^4^ (Fig. S7E). The V125–P132 and K141-R166 motifs in DDIT4L were the potential major binding sites for ATP5A (Fig. 1M). We also predicted potential DDIT4L binding motifs within ATP5A (Fig. S7F). GST pull-down assay pinpointed the K132–I158 motif of ATP5A as the primary binding domain for DDIT4L (Fig. S7G). Furthermore, the interaction between DDIT4L and ATP5A was disturbed by the synthesized TAT-ATP5A^K132-I158^ peptide (Fig. S7H). The structural analysis showed that the V125–P132 domain of DDIT4L was a functional domain for ATP5A binding (Fig. 1N). In summary, DDIT4L and ATP5A could interact each other via the specific motifs.

Next, we examined the possible role of DDIT4L in mitochondrial function. *TOMM4*0 mRNA levels was elevated more substantially than *TOMM22* or *TOMM2*0 mRNA levels in GBM (Fig. S8A and S8B), suggesting that DDIT4L was likely to interact with the endogenous single TOM40 protein in GBM cells. DDIT4L overexpression in U87MG cells decreased the cellular ATP content and mitochondrial membrane potential (Fig. S8C and S8D). As expected, DDIT4L reduced both the basal and ATP synthase-linked oxygen consumption rates (OCRs) (Fig. 1O), induced mitochondrial fusion and reduction in the number of cristae in primary GBM cells (Fig. 1P; Fig. S8E). DDIT4L reduced mitochondrial cytochrome c and concurrently increased its cytoplasmic level in primary GBM and U87MG cells, accompanied by elevated cleaved caspase-3 (Fig. S8F and S8H). The TUNEL assay showed that DDIT4L induced apoptosis in primary GBM cells (Fig. S8G).

In the patient-derived and U87MG cell orthotopic mouse model of GBM, lentivirus-mediated DDIT4L overexpression reduced the tumor size and extended the lifespan (Fig. S9A–S9F). The TAT-DDIT4L^V125–P132^ peptide, but not TAT- DDIT4L^K141–R166^ peptide, reduced the proliferation of tumor cells in a dose-dependent manner (Fig. S9G). The TAT-DDIT4L^V125–P132^ peptide also reduced the ATP synthase-linked OCRs in tumor cells (Fig. S9H). Importantly, local delivery of the TAT-DDIT4L^V125–P132^ peptide via subcutaneous minipumps significantly reduced the tumor size and prolonged the lifespan (Fig. 1Q–S). Together, exogenous excessive DDIT4L and the TAT-DDIT4L^V125–P132^ peptide inhibited GBM tumor growth and prolonged the animals’ lifespan.

Altogether, these findings provide novel insights into the relationship between mitochondrial function and cell apoptosis during GBM oncogenesis.

## CRediT authorship contribution statement

**Pu You:** Writing – original draft, Methodology. **Zeng-Xin Qi:** Writing – original draft, Methodology. **Hao Luo:** Writing – original draft, Methodology. **Bing Cai:** Methodology. **Xu-Ye Hu:** Methodology. **Jing Pan:** Methodology. **Pan-Pan Gan:** Methodology. **Xin-Ying Zeng:** Methodology. **Ri-Jing Liao:** Methodology. **Qisheng Tang:** Methodology. **Ye Yu:** Methodology. **Zhen-Ning Zhang:** Methodology. **Bojie Yang:** Resources, Conceptualization. **Liang Chen:** Writing – review & editing, Writing – original draft, Funding acquisition, Conceptualization. **Xu Zhang:** Writing – review & editing, Writing – original draft, Funding acquisition, Conceptualization. **Kai-Cheng Li:** Writing – review & editing, Writing – original draft, Funding acquisition, Conceptualization. **Ying Mao:** Writing – review & editing, Writing – original draft, Funding acquisition, Conceptualization.

## Ethics declaration

Generation of PDX model was conducted with the approval of the Zhangjiang Laboratory Institutional Review Board. The ethical approval for collection of human tumor tissues was approved by the Huashan Hospital of Fudan University (SHIRB, KY2015-256). Written informed consent was obtained from all participants.

## Data availability

The datasets generated and/or analysed during the current study are available in the TCGA program (https://portal.gdc.cancer.gov) and CGGA program (https://www.cgga.org.cn/).

## Funding

This study was supported by 10.13039/501100012166National Key R&D Program of China, MOST (No. 2023YFC2510000), Grants of Shanghai Research Center for Brain Science and Brain-Inspired Intelligence (China) (No. A182A01C1, LKC), Talents Program of Shanghai Municipal Health Commission (CL), Shanghai Municipal Science and Technology Major Project (China) (No. 2018SHZDZX01), Zhangjiang Laboratory, and Shanghai Center for Brain Science and Brain-Inspired Technology.

## Conflict of interests

The authors declare no competing interests.
