# Supplementary material for: The DDIT4L-TOM40-ATP5A pathway suppresses glioblastoma oncogenesis
Source: Genes Dis. 2026 Mar 4;14(1):102121. doi: 10.1016/j.gendis.2026.102121 (PMC13571239; doi:10.1016/j.gendis.2026.102121)
Supplement: Supplementary file 1 [file mmc1.docx]

**The DDIT4L-TOM40-ATP5A pathway suppresses glioblastoma oncogenesis**

**Materials and Methods**

**Human Tumor and cell Cultures and PDX model**

Human tumor tissue collection and PDX model generation was conducted with the approval of the Huashan Hospital of Fudan University and the Zhangjiang Laboratory Institutional Review Board (SHIRB, KY2015-256). Informed consent was obtained from all subjects involved in this study. GBM and LGG tissues were obtained from newly diagnosed patients with the following clinical characteristics: hGBM21359, 71 year old male; hGBM11879, 68 year old male; hGBM11271, 42 year old male; hGBM12913, 64 year old female; hGBM79150, 53 year old female; hGBM21686, 28 year old female; hLGG14023, 38 year old male, grade III; hLGG11002, 53 year old male, grade III; hL21845, 56 year old male, grade III; hLGG12101, 39 year old female, grade II; hLGG21823, 24 year old male, grade II.

Patient-derived primary GBM cells from hGBM11879 and hGBM11271 were maintained in neurosphere medium (DMEM/F12, ThermoFisher, 11330032), supplemented with B27 (0.5X, ThermoFisher, 17504044), N2(0.5X, ThermoFisher, 17504048), bFGF (20 ng/ml, Peprotech, 100-18B) and EGF (20 ng/ml, Peprotech, 100-47), BSA (0.5mg/ml, Sigma-Aldrich, A9418).

Intracranial transplantation of primary GBM cells to establish tumor xenografts was performed as described^1^. Briefly, 48 hr after lentiviral infection, 2×10^5^ primary GBM cells were implanted into the left CPU region (from bregma, cells were injected 2.0 mm lateral, 0 mm caudal, 2.75 mm ventral) of male Nod-SCID or Nude mice. The 8-week old male littermates were used for transplantation and randomized to receive primary GBM cells expressing DDIT4L or control vector. For the survival experiments, animals were maintained until manifestation of neurological signs or for 120 days post-transplantation. To monitor tumor growth, mice transplanted with primary GBM cells, which stably expressed firefly luciferase, were monitored by bioluminescence imaging longitudinally using the IVIS100 bioluminescence imaging system. To analyze the effect of peptide on the growth of GBM cells in vivo, osmotic pumps (ALZET®, 1002) were implanted subcutaneously to direct deliver TAT-DDIT4L^V125-P132^ peptide or scrambling peptide (dissolved in PBS) to the injection site via brain infusion kit (ALZET®) a week after intracranial tumor cell injection.

**Immunostaining**

Frozen brains were sectioned to 8-15 μm thickness. Sections were blocked in 10% normal donkey serum prepared in PBS and 0.3% TritonX-100 for 1 hour. Sections were incubated overnight at 4°C with the following primary antibodies: DDIT4L (1:500; Sigma; SAB1408239); DDIT4L (1:200; Invitrogen; PA5-48874); SOX2 (1:200; STEMCELL; 60055); Olig2 (1:200; Santa Cruz; sc-48817); GFAP (1:200; Sigma; G9269); Nestin (1:500; STEMCELL; 60091); CD133 (1:200; Chemicon; MAB4310); ATP5A (1:500; Abcam; ab14748); HIF1α (1:100; Sigma; SAB2702132) and CD31 (1:50; Sigma; SAB5500059). On the next day sections were incubated with biotinylated secondary antibodies, followed by signal amplification and visualization with the avidin–biotin complex (ABC) system and DAB substrate (Vector Laboratories). Leica SP8 microscope was used for imaging.

**Proximity Ligation Assay (PLA)**

Adherent cell deposited on glass slides have reached the desired confluence and pre-treated with 4% PFA for 15 mins at room temperature. Using 1×Blocking solution to block and incubate the slides in a humidity chamber for 60 minutes at 37°C. Slides were incubated overnight at 4°C with the following primary antibodies: Myc (1:2000; Sigma; C3956); HA (1:2000; Sigma; H3663). On the next day the slides were incubated with PLUS and MINUS PLA probes (1:5; Sigma; DUO92001 and DUO92005) for 1 hour at 37°C. Ligase the slides with 1X Ligation Buffer (1:40; Sigma; DUO92008) and incubate the slides for 30 minutes at 37°C. The slides were incubated with Amplification-Polymerase solution (1:80; Sigma; DUO92008) for 100 mins at 37°C. Mount the slides with a cover slip using a minimal volume of Duolink In Situ Mounting Medium with DAPI (Sigma; DUO82040). Leica SP8 microscope was used for imaging.

**Quantification of Mitochondrial Morphology**

Quantification of mitochondrial morphology followed the method reported before. In brief, the quantification was scored from more than 150 cells with three replicates, and it was done by a person who was blinded to the treatment of the samples. Morphology was classified as follows: “fragmented”, the majority of mitochondria were less than 5μm; “intermediate”, the majority of mitochondria were more than 5μm; and “tubular”, highly interconnected mitochondria with fewer than 4-5 free ends.

**Immunogold TEM**

Sections (90 nm) were mounted on 100 mesh nickel Formvar-carbon coated grids, incubated with PBS containing 0.1M Glycine (Sigma-Aldrich) and 1% Bovine Serum Albumin (Sigma-Aldrich). Sections were incubated with myc antibody over night at 4°C, washed extensively with PBS (5 × 10 min) and exposed to secondary antibody gold (15nm, Sigma-Aldrich) for 2 hours at RT. Lastly, specimens were stained with 2% uranyloxalicacetate (pH=7, Sigma-Aldrich) for 5 min at RT and Rinse grids extensively in H_2_O and view at 80 kV in a transmission electron microscope. In total 50 fields containing 1-3 cells were used for a quantitative analysis of the samples. The labeling ratio were determined by counting the intracellular gold particles and gold particles outside or inside the mitochondria membrane.

**Western blots, Antibodies, and siRNA Transfection**

The tissue and cells were lysed in ice-cold buffer (50 mM Tris, 150 mM NaCl, 0.1% Triton-100, 10% glycerol, 0.5 mg/ml BSA and protease inhibitors). After measuring protein concentration, western blot samples were prepared for SDS-PAGE. Antibodies purchased from Santa Cruz include Tom20 (sc-11415), from Cell Signaling include cleaved Caspase 3 (#9661s), phospho-S6 (#9234s), S6 (#2708s), phosphor-Akt (#9271s) from Sigma include DDIT4L (SAB1408239 or SAB2100546), HIF1α (SAB2702132), HA-tag (H3663), myc-tag (C3956), Flag-tag (F3165), from Proteintech include myc-tag (60003-2-Ig), GAPDH (10494-1-AP), from Abcam include ATP5A (ab14748), ATP5B (ab14730), from Amersham pharmacia include GST (#27-4577). Random shuffle sequence for control and a DDIT4L siRNA (DDIT4L-homo-620 sequence: GUGUGAUUCUAGCGUCGUA) from GenePharma was used to knockdown experiments in GBM cell lines. Lipofectamine® 3000 transfection reagent (Thermofisher, L3000015) was used for siRNA transfection according to manufacturer’s instructions.

**Immunoprecipitation and GST pull-down**

The GBM primary cells (hGBM11879), U87MG, COS7, HEK293 and HEK293T cells were lysed in ice-cold buffer (50 mM Tris, 150 mM NaCl, 0.1% Triton-100, 10% glycerol, 0.5 mg/ml BSA and protease inhibitors). The suspended lysate was immunoprecipitated with antibodies for 1 hour at 4°C and then with Protein G-Agarose for Rb or Mo antibodies overnight at 4°C. Immunoprecipitates were collected and aspirated. The sepharose was resuspended in RIPA buffer without SDS, washed at least 3 times, and incubated in SDS buffer for 20 min at 60°C. Then the immunoblotting was processed. The immunoblotting band in U87MG cells was analyzed for mass spectrometry. The GST-fused protein (5 mg) was incubated with the cell lysate at 4°C overnight. Then, the GST-fused protein was precipitated with 10 mL of Glutathione-Sepharose beads at 4°C for 2 hours. The precipitant was washed, denatured and prepared for immunoblotting.

**Homology Modeling and *in-silico* Docking hDDIT4L into hTOM40 and hATPase**

The homology model of human DNA damage inducible transcript 4-like (hDDIT4L) dimer was created based on the crystal structure of human REDD1, a hypoxia-induced regulator of mTOR (3LQ9, 2Å) using Modeller. The sequence of hDDIT4L was retrieved from the Uniport (entry: [Q9NX09](https://www.uniprot.org/uniprot/Q9NX09)). According to the secondary structure information of the template, the sequence alignment was adjusted manually to obtain a more reasonable alignment. The homology model of human TOM40 was created from structure of the TOM core complex from neurospora crassa. The homology model of human ATP synthase was created based on the ground state structure of F1-ATPase from bovine heart mitochondria (2JDI, 1.9Å). The constructed homology model was checked and validated by the program Procheck. hDDIT4L/hTOM40 and hDDIT4L/hATPase interaction models were predicted by ZDOCK. 2000 possible binding poses were obtained and clustered, and then the selected best pose was optimized by DESMOND using the default parameters.

**Assay for Cellular ATP Levels**

The U87 cells were seeded in 96-well plates at 5 x 10^4^ cells per well and transfected with DDIT4L and control vector for 30 hours in triplicate. ATP levels were measured using the ATPlite 1step general assay (Perkinelmer, 6016739); luminescence was read using Spectramax^@^ iD3 (Molecular Devices). Readings were normalized to cell number.

**Mitochondrial Membrane Potential Measurement**

The JC-10 mitochondrial membrane potential assay kit was purchased from Abcam (112134) and the assay was performed according to the manufacturer’s protocol. Cells were transfected with DDIT4L and control vector for 30 hours, then measuring of mitochondrial membrane potential with Spectramax^@^ iD3 (Molecular Devices).

**Oxygen Consumption Rate (OCR)**

OCR was measured by Seahorse Bioscience instrument (XF24/XF96, Agilent) with 80-90% confluent cells according to the manufacturer’s protocol. Briefly, on the day following cell seeding, cells were equilibrated for 1 hour in a 37°C incubator lacking CO_2_. Oxygen concentration in media was measured at basal conditions and after sequential addition of compounds and peptides as indicated in corresponding figure legends. Concentration of compounds and peptides used were: oligomycin A (1 μM), Fccp (1 μM), a mixture of rotenone (500 nM) and antimycin A (500 nM), TAT-DDIT4L^V125-P132^ or scramble peptides as described in specific figure legend. A minimum of three wells were utilized per condition to calculate OCR.

**Peptide Synthesis and Preparation**

The peptides used in this study were prepared by solid-phase peptide synthesis (SPPS) using standard Fmoc (9-fluorenylmethoxycarbonyl) chemistry. The synthesis was performed on a Rink amide resin to generate C-terminal amidated peptides.

For each cycle, the Fmoc protecting group was removed by treating the resin with 20% piperidine in DMF. Subsequent amino acid couplings were carried out using a 4-fold molar excess of Fmoc-protected amino acids activated with HATU and N, N-Diisopropylethylamine (DIEA) in DMF. After completion of the chain assembly, the peptides were cleaved from the resin and simultaneously deprotected using a trifluoroacetic acid (TFA) cocktail containing TFA/H2O/triisopropylsilane (95:2.5:2.5, v/v/v) for 3 hours at room temperature.

The crude peptides were then precipitated and washed with cold diethyl ether, dissolved in water, and purified by reverse-phase high-performance liquid chromatography (RP-HPLC) on a C18 column. The molecular weights and purities (>95%) of the final products were confirmed by analytical HPLC and mass spectrometry. The purified peptides were lyophilized and stored at -80℃ until use.

**Bioinformatics Analysis**

TCGA diffuse glioma database including RNA-seq, simple somatic mutation and patient survival were downloaded from the TCGA Research Network by R Studio Version 1.2.5033 with packages TCGA biolinks. Gene expression of DDIT4L and other genes in the TCGA diffuse glioma database were processed using publicly available tools including the biostatistics program R Studio Version 1.2.5033 with packages gplots, DT, dplyrand Summarized Experiment. DDIT4L expression level and clinical data of Chinese Glioma Genome Atlas were downloaded from online database (CGGA, http://www.cgga.org.cn). In addition, DDIT4L expression level of Gravendeel dataset and Rembrandt dataset were obtained from the online Visualization Tools for Glioma Datasets (GlioVis, http://gliovis.bioinfo.cnio.es).

**Cell Growth and Viability Assays**

Cells were seeded in 6-well plates, transfected with DDIT4L-myc and control plasmids or DDIT4L and control RNAi or treated with TAT-DDIT4L^V125-P132^ peptide or scrambling peptide, then harvesting cells at 0, 1, 2, 3 days, staining with trypan blue (0.4%, Thermofisher, T10282). Cell number and viability were measured by automated cell counter Cellometer^®^Auto 1000 (Nexcelom Bioscience) following the manufacturer’s instructions.

**Neurosphere assay**

For the sphere assay, 5000 dissociated primary cells were seeded into one well of a 6 well Ultra-Low attachment plate (Life Sciences Costar®, 3471) and grown in NSC medium for 2 weeks and imagined every other day. A picture of the whole well was taken with a Zeiss widefield microscope and the neuroshperes were counted and analyzed with ImageJ. The assay was repeated at least three times for all cell lines and the significance was analyzed with a One-Way ANOVA test.

**Flow Cytometry**

Flow cytometry was performed on a BD LSRFortessa™ X-20 cell analyzer (BD Biosciences) and analyzed using FlowJo software (Tree Star). The primary cells were incubated for 10 min at 37°C in 10 ml of DPBS (Thermofisher, 14190144) containing 0.25% trypsin (Thermofisher, 25300). The suspension was centrifuged at 800g for 5 min at 4°C. The pellet was re-suspended in DPBS and centrifuged again at 800g for 5 min at 4°C. The pellet was re-suspended in 1 ml paraformaldehyde (4%, pH=7.4) and stayed for 10 min at 4°C. The suspension was centrifuged at 800g for 5 min at 4°C. The pellet was re-suspended in DPBS and centrifuged again at 800g for 5 min at 4°C. The pellet was re-suspended in 0.1% Triton X-100 for 10 min at 4°C. The suspension was centrifuged at 1200g for 10 min at 4°C. The pellet was re-suspended in PBA (PBS consisting of 1% BSA, 0.1% Sodium azide) containing primary antibodies incubated for 1 h at 4°C. The suspension was centrifuged at 1200g for 10 min at 4°C. The pellet was re-suspended in PBA containing secondary antibodies incubated for 30 min at 4°C. The suspension was ready for testing, fluorescence-minus-one control-based gating.

**Plasmid Construction**

All primers and oligonucleotides used for the construction of plasmids expressing DDIT4L and DDIT4L truncations, ATP5A and ATP5A truncations, ATP5B, GST-DDIT4L, GST-ATP5A are listed in Table S2.

The cDNA of human DDIT4L was cloned into pcDNA3.1/myc-his(+)A vector for the expression of DDIT4L-myc. The plasmids expressing DDIT4LΔ1-myc, DDIT4LΔ2-myc and DDIT4LΔ3-myc were modified from the DDIT4L-myc plasmid using KOD-plus Mutagenesis Kit (Toyobo). The cDNA of human TOM20, TOM22, TOM40, ATP5A and ATP5B was constructed in a modulated pcDNA3.1/HA-his(+)A vector as a HA tag replaced the myc tag in the pcDNA3.1/myc-his(+)A vector. The plasmids expressing ATP5AΔ1-HA, ATP5AΔ2-HA, ATP5AΔ3-HA, ATP5AΔ4-HA and ATP5AΔ5-HA were modified from the ATP5A-HA plasmid using KOD-plus Mutagenesis Kit (Toyobo). The cDNA of human DDIT4L and human ATP5A following a HA tag was cloned into a pGEX vector for the construction of the GST-DDIT4L and GST-ATP5A-HA plasmids.

**Preparation of the GST-Fused Proteins**

The GST-fused proteins were expressed in Escherichia coli BL21. The bacteria were grown in 23YTA media and the protein expression was induced by 1 mM Isopropyl b-D-thiogalactopyranoside (IPTG). Then the bacteria were centrifuged, resuspended and sonicated for the protein release. The proteins were purified using Glutathione-Sepharose beads (GE Healthcare), concentrated and quantified before use.

**Transmission electron microscope (TEM)**

The primary cells (5×10^5^) were collected after transfected with 3 μg DDIT4L or control plasmid for 48h. Then, the cells were first fixed in 2.5% Glutaraldehyde buffered in 0.1M PBS for 2h. The samples were post-fixed with 1% OsO_4_ in the dark for 1.5h. The samples were dehydrated through alcohol with gradient concentrations (30, 50, 70, 80, 95, and 100%) and further acetone dehydration twice (100%). Finally, the samples were treated with acetone: Epon812 (1:1) for 2h, and treated with Epon812 (100%) overnight. Then, the ultra-thin sections (70 nm) were obtained, stained with Uranium acetate and Lead citrate, and photographed under a TEM at 80kV (FEI Tecnai G2 Spirit). Mitochondria were manually segmented, the area and diameter measured in Fiji software by adding up the voxel.

**Mass Spectrometry (MS)**

For MS analysis, the in-gel digestion was performed using the following protocol. To reduce disulfide bonds, the strip was performed in 1% 1, 4-dithiotreitol (DTT) in SDS equilibration buffer [50 mM Tris-Cl (pH=8.8), 6 M urea, 30% glycerol, 2% SDS and bromophenol blue] for 15 min. This step was followed by alkylation of the free sulhydryl groups by 2.5% iodoacetamide in a SDS equilibration buffer for another 15 min in the dark at room temperature. Then the strip was cut into 18 gel sections (each section about 1.0 cm in length). Each gel section was washed three times alternately with acetonitrile and 100 mM ammonium bicarbonate. During the last wash the gel slices were incubated in 100 mM ammonium bicarbonate for 15 min at temperature 4°C. The gel slices were dried by vacuum centrifugation and allowed to swell in a 50 μl trypsin solution containing trypsin (20 μg/ml) and ammonium bicarbonate (50 mM) at 4°C for 45 min. After adding another 50 μl of trypsin solution, the gel slices were kept at 37°C for 20 h. The supernatant was transferred to another vial, and the gel slices were extracted for 15 min three times by 0.1% formic acid in 60% acetonitrile. The recovered peptide solutions were dried by vacuum centrifugation and desalted and cleaned using a Ziptip (Millipore, Corp., Bedford, MA).

The peptide mixtures from each section of the strip were separated by Reverse phase HPLC (RP-HPLC) followed by tandem mass analysis. RP-HPLC was performed on a surveyor LC system (Thermo Finnigan, San Jose, CA). The C18 column (RP, 180 μm × 150 mm) was obtained from Column Technology Inc. (Fremeont, CA,). The pump flow was split 1:120 to achieve a column flow rate of 1.5 μl/min. Mobile phase A was 0.1% formic acid in water, and mobile phase B was 0.1% formic acid in acetonitrile. The tryptic peptide mixtures were eluted using a gradient of 2-98% B over 180 min.

The MS was performed on a LTQ linear ion trap mass spectrometer (Thermo Finnigan, San Jose, CA) equipped with an electrospray interface and operated in positive ion mode. The capillary temperature was set to 170°C and the spray voltage was at 3.4 kV. Normalized collision energy was at 35%. Automatic gain control was used to obtain maximal signal of each scan. The mass spectrometer was set so that one full MS scan was followed by ten MS/MS scans on the 10 most intense ions. Dynamic Exclusion was set at repeat count 2, repeat duration 30 s, and exclusion duration 90 s.

The acquired MS/MS spectra were searched against the IPI rat database using BioWorks 3.0 software (Thermofinnigan) on an 8 node Dell PowerEdge 2650 cluster. An accepted SEQUEST result had a ΔCn score of at least 0.1 (regardless of charge state), a value known for high confidence in a SEQUEST search. All output results were combined together using homemade software named Build Summary to delete the redundant data. To make sure that the MS/MS spectrum was of good quality with fragment ions clearly above baseline noise, we referred to the parameters reported in previous studies and applied stricter criteria for peptide identification. Peptides were validated after meeting the following criteria. The SEQUEST cross-correlation score must be ≥1.9 for a +1 tryptic peptide, ≥2.2 for a +2 tryptic peptide and ≥3.75 for a +3 tryptic peptide. In addition, ΔCn cutoff values were ≥0.1 and the SP rank of the peptides ≤4.

**Magnetic resonance imaging (MRI)**

All in vivo MRI measurements were performed on a 7.0 Tesla small animal MR system (Biospec, Bruker Biospin). Mice were anesthetized by inhalation narcosis using 0.5% to 1.5% isoflurane (RWD), pressurized oxygen. Core body temperature was maintained at 37°C. Respiration rate and temperature were monitored using a remote monitoring system (Model RM400S, RWD Inc.). T2-weighted images were acquired with the same slice geometry (slice thickness = 500 µm, 25 coronal slices covering a brain region of 12.5 mm (approx. Bregma 4 to -8.5mm)). Tumor regions were manually segmented and the volume calculated in Fiji software by adding up the voxel volumes.

**Statistical Analysis**

The data are presented as mean ± SEM. Sample number (n) values are indicated in figures, figure legends or results section. Two groups were compared by a two-tailed, unpaired Student’s t test. Comparisons between two groups with multiple time were performed by a two-way ANOVA with Bonferroni’s post hoc test. Survival analysis was performed using the Kaplan-Meier method and comparisons were done using the log-rank test.

Statistical analysis was performed using PRISM (GraphPad Software). Differences were considered significant at p < 0.05 (*p < 0.05, **p < 0.01, ***p < 0.001)

**Supplementary Figure legends**

**Supplementary Figure 1. DDIT4L is expressed in aggressive glioma and suppresses tumorsphere formation.** (A) The upregulated genes from the TCGA database via overlapping GBM & LGG, IDH wild-type & mutation GBM, and mesenchymal & proneural type of GBM. (B) Top 10 upregulated genes are identified in the GBM & LGG comparative analysis. (C) Boxplot of the *DDIT4L* expression in GBM & LGG from the TCGA database. (D) Boxplot of the *DDIT4L* expression in IDH wild-type & mutation GBM, and mesenchymal & proneural type of GBM from the TCGA database. (E) Boxplot of the *DDIT4* expression in GBM & LGG from the TCGA database. Significance testing was done with Mann-Whitney *U* test. (F) *DDIT4L* expression level in Gravendeel dataset and Rembrandt dataset were obtained from the online tool GlioVis (Visualization Tools for Glioma Datasets). (G) The CGGA database demonstrated the distinct survival time of glioma patients with different grades. Kaplan-Meier survival analysis of patients with different WHO grades of glioma stratified by the *DDIT4L* expression. Median *DDIT4L* expression was used for the stratification into DDIT4L-high and DDIT4L-low tumors. (H) The TCGA database demonstrated the similar survival time of GBM patients with different expression level of *DDIT4L*. Kaplan-Meier survival analysis of patients with GBM stratified by the *DDIT4L* expression. Median *DDIT4L* expression was used for the stratification into DDIT4L-high and DDIT4L-low tumors.

**Supplementary Figure 2. Co-expression of DDIT4L and glioma-initiating markers.** (A) DDIT4L partially co-expressed with Nestin and CD133 by the flow cytometry. (B) Double-immunostaining of DDIT4L and SOX2 in GBM tissues. Scale bar = 100 μm.

**Supplementary Figure 3. Hypoxia up-regulates DDIT4L expression, DDIT4L Localizes in hypoxia-sensitive GBM cells.** (A) DDIT4L and HIF1α expression increased with the aggression of glioma from grade II to grade IV. (B) DDIT4L increased in GBM cells after exposure of hypoxia (1% O_2_). (C) Double-immunostaining of DDIT4L and HIF1α, or DDIT4L and CD31 in GBM. Scale bar = 50 μm. (D) DDIT4L partially co-expressed with HIF1α, not with CD31 by the flow cytometry. (E) Transfected DDIT4L plasmid apparently inhibits U87MG cell proliferation 3 days after transfection. (F) Knockdown of DDIT4L by RNAi significantly enhances U87MG cell proliferation 3 days after transfection. (G, H) Lentiviral infection of DDIT4L apparently inhibits the tumorsphere formation in the primary GBM cells. Scale bar = 200 μm.

**Supplementary Figure 4. DDIT4L binds to TOM40.** (A) Coomassie blue staining shows a band of molecular weight ~40 kDa in the myc-antibody-precipitated proteins from U87MG cells transfected the DDIT4L-myc plasmid. Co-IP shows that TOM40 (~40 kDa) in DDIT4L-precipitated proteins from GBM tissue (n = 3). (B) In the lysate of HEK293 cells co-transfected with plasmids expressing TOM40-HA and DDIT4L-myc, TOM40-HA is found in the proteins precipitated with myc antibodies (n = 3). (C) Co-IP shows that TOM20, TOM22 and TOM70 were not in myc-antibody precipitated proteins from HEK293 cells expressing DDIT4L-myc (n = 3 for all experiments). (D) Possible docking site of DDIT4L in the TOM40 using a combination of homology modeling and *in-silico* docking. (E) The selected DDIT4L (pink cartoon)/TOM40 (gray surface) model was derived from a cluster analysis of 2000 possible binding models based on ZDOCK scores and optimized by Zrank evaluation. The S96-C106P loop of DDIT4L, but not V125-P132 and K144-R106, might interact directly with TOM40.

**Supplementary Figure 5. Subcellular distribution of DDIT4L.** (A) DDIT4L did not co-localize with mitochondria marked by mitochondria tracker (Mitotracker) after transfection of DDIT4L. Scale bar = 10 μm. (B) DDIT4L co-localized with mitochondria marked by TOM40 after co-transfection of DDIT4L and TOM40. Scale bar = 10 μm. (C) Quantification shows that the ratio of DDIT4L positive and Mitotracker positive cells/DDIT4L positive cells.

**Supplementary Figure 6. Co-localization of DDIT4L and Lamp1 or Mitotracker.** (A) Transfected DDIT4L co-localized with Lamp1, not co-localize with Mitotracker, while co-transfected DDIT4L and TOM40, the DDIT4L mainly co-localized with Mitotracker, not co-localize with Lamp1 in the HEK293T cells. Scale bar = 10 μm. (B) Quantification shows that the ratio of DDIT4L positive and Lamp1 positive cells/DDIT4L positive cells.

**Supplementary Figure 7. DDIT4L interacts with ATP5A.** (A) Coomassie blue staining shows a band of molecular weight ~50 kDa in the myc-antibody-precipitated proteins from U87MG cells transfected the DDIT4L-myc plasmid. Co-IP shows that ATP5A (~50 kDa) in myc-antibody-precipitated proteins from U87MG cells expressing DDIT4L-myc (n = 3). (B) In the lysate of GBM primary cells (hGBM11879) co-transfected with plasmids expressing ATP5A-HA or ATP5B-HA and DDIT4L-myc, both ATP5A-HA and ATP5B-HA are found in the proteins precipitated with myc antibodies (n = 3). (C) The GST pull-down assay shows that GST-DDIT4L protein interacts with ATP5A-HA (n = 3). (D) Co-IP with two purified proteins, GST-ATP5A and GST-DDIT4L, shows the direct interaction between ATP5A and DDIT4L (n = 3). (E) Possible docking site of DDIT4L in the ATPase using a combination of homology modeling and in-silico docking. In this model, we screened out S96-C106, V125-P132 and K141-R166 motifs in DDIT4L as the possible binding domains for ATP5A. (F) Five motifs (V118-V131, K132-I158, I179-G192, L471-A486, G491-I504) of ATP5A had been predicted as the binding domains for DDIT4L using a combination of homology modeling and *in-silico* docking. (G) The GST pull-down assay shows that GST-DDIT4L protein interacts with ATP5A-HA, ATP5AΔ3-HA, ATP5AΔ4-HA and ATP5AΔ5-HA, but the interaction is decreased in ATP5AΔ1-HA and ATP5AΔ2-HA in HEK293T cells (Δ1: deleting region of V118-V131, Δ2: deleting region of K132-I158, Δ3: deleting region of I179-G192, Δ4: deleting region of L471-A486, Δ5: deleting region of G491-I504) (n = 3). (H) The TAT-ATP5A^K132-I158^ peptide apparently reduced the interaction of DDIT4L and ATP5A in the transfected HEK293T cells (n = 3).

**Supplementary Figure 8. DDIT4L effect on mitochondrial function and cell apoptosis.** (A) The level of *TOM40* mRNA was increased significantly, while level of *TOM22* mRNA had no change and *TOM20* mRNA was decreased in GBM tissues. (B) The CGGA dataset demonstrated that *TOM40* expression level was increased in WHO grade III and IV when compared to that in Grade II gliomas. (C) Decreased cellular ATP content in tumor cells transfected with the plasmid expressing DDIT4L. (D) Decreased mitochondrial membrane potential in tumor cells transfected with the DDIT4L plasmid. (E) Mitochondrial morphology is shown by a mitochondrial marker Mitotracker in the GBM cells transfected with DDIT4L plasmid. Scale bar = 10 μm. DDIT4L apparently increases the tubular-like mitochondria in cells. (F) The protein level of cytochrome c and cleaved caspase 3 in the cytoplasm are increased via isolating mitochondria from cytoplasm of primary GBM cells transfected with the plasmid expressing DDIT4L (n = 3). (G) The TUNEL assay shows that DDIT4L induces cell apoptosis (green) in primary GBM cells (DAPI, blue). Scale bar = 50 μm. (H) Isolated mitochondria from cytoplasm in U87MG cells, the cytochrome c and cleaved caspase 3 significantly increase in cytoplasm after transfected DDIT4L (n = 3).

**Supplementary Figure 9. DDIT4L and TAT-DDIT4L^V125-P132^ suppress oncogenesis of GBM.** (A) Bioluminescence imaging of GBM xenografts derived from patient tumor cells expressing the luciferase and the lentivirus vector control or lentivirus DDIT4L show that DDIT4L inhibit tumor growth. Representative images are captured from animals on day 70 after cells intracranial transplantation. (B) Total Flux of photon emission is decreased by DDIT4L (n = 10 per group). (C) Kaplan-Meier survival analysis of mice with GBM xenografts after the transfection with lentivirus expressing DDIT4L shows that overexpressing of DDIT4L prolong the survival time significantly (n = 10 per group). (D) MRI and bioluminescence imaging of GBM xenografts derived from U87MG cells expressing luciferase and with vector control or DDIT4L. Images from animals on day 28 after cells intracranial transplantation are shown. Scale bar = 1 mm. (E) Transfected DDIT4L apparently decreases tumor volume of GBM xenografts derived from U87MG cells (n = 6). (F) Kaplan-Meier survival analysis of mice with GBM xenografts after transfected DDIT4L. (G) TAT-DDIT4L^V125-P132^, but not TAT-DDIT4L^K144-R166^, produces an inhibitory effect on the U87MG cell proliferation in a dose-dependent manner (n = 3). (H) Seahorse Mito Stress Kit assay on U87MG cells showed that TAT-DDIT4L^V125-P132^-induced OCRs decreased when compared with the scrambled peptide-induced OCRs (n = 3).
