# Supplementary material for: The DDIT4L-TOM40-ATP5A pathway suppresses glioblastoma oncogenesis
Source: Genes Dis. 2026 Mar 4;14(1):102121. doi: 10.1016/j.gendis.2026.102121 (PMC13571239; doi:10.1016/j.gendis.2026.102121)
Supplement: Supplementary file 2 [file mmc2.docx]

**Table S1. Overlapping genes up-regulated in three paired classifications from the TCGA database**

| **SYMBOL** | **GENE ID** | **Full name** | **Order** |
| --- | --- | --- | --- |
| TCEA3 | 6920 | transcription elongation factor A3(TCEA3) | 1 |
| DDIT4L | 115265 | DNA damage inducible transcript 4 like(DDIT4L) | 2 |
| FAM86B2 | 653333 | family with sequence similarity 86 member B2(FAM86B2) | 3 |
| C6orf141 | 135398 | chromosome 6 open reading frame 141(C6orf141) | 4 |
| PDLIM4 | 8572 | PDZ and LIM domain 4(PDLIM4) | 5 |
| TCTEX1D1 | 200132 | Tctex1 domain containing 1(TCTEX1D1) | 6 |
| TNFRSF11B | 4982 | TNF receptor superfamily member 11b(TNFRSF11B) | 7 |
| ANXA2P1 | 303 | annexin A2 pseudogene 1(ANXA2P1) | 8 |
| FMOD | 2331 | fibromodulin(FMOD) | 9 |
| POSTN | 10631 | periostin(POSTN) | 10 |
| KCTD14 | 65987 | potassium channel tetramerization domain containing 14(KCTD14) | 11 |
| STEAP1 | 26872 | STEAP family member 1(STEAP1) | 12 |
| LIF | 3976 | leukemia inhibitory factor(LIF) | 13 |
| KRT7 | 3855 | keratin 7(KRT7) | 14 |
| STAC | 6769 | SH3 and cysteine rich domain(STAC) | 15 |
| HFE | 3077 | hemochromatosis(HFE) | 16 |
| ABCC3 | 8714 | ATP binding cassette subfamily C member 3(ABCC3) | 17 |
| SGMS2 | 166929 | sphingomyelin synthase 2(SGMS2) | 18 |
| EVC2 | 132884 | EvC ciliary complex subunit 2(EVC2) | 19 |
| COL8A1 | 1295 | collagen type VIII alpha 1 chain(COL8A1) | 20 |
| HS3ST3B1 | 9953 | heparan sulfate-glucosamine 3-sulfotransferase 3B1(HS3ST3B1) | 21 |
| IL7 | 3574 | interleukin 7(IL7) | 22 |
| CHI3L1 | 1116 | chitinase 3 like 1(CHI3L1) | 23 |
| SAA1 | 6288 | serum amyloid A1(SAA1) | 24 |
| ARSJ | 79642 | arylsulfatase family member J(ARSJ) | 25 |
| APOBEC3D | 140564 | apolipoprotein B mRNA editing enzyme catalytic subunit 3D(APOBEC3D) | 26 |
| CSTA | 1475 | cystatin A(CSTA) | 27 |
| MOXD1 | 26002 | monooxygenase DBH like 1(MOXD1) | 28 |
| FBXO39 | 162517 | F-box protein 39(FBXO39) | 29 |
| LCTL | 197021 | lactase like(LCTL) | 30 |
| DMRTA2 | 63950 | DMRT like family A2(DMRTA2) | 31 |
| GPX8 | 493869 | glutathione peroxidase 8 (putative)(GPX8) | 32 |
| ETV7 | 51513 | ETS variant 7(ETV7) | 33 |
| PTX3 | 5806 | pentraxin 3(PTX3) | 34 |
| SAA2 | 6289 | serum amyloid A2(SAA2) | 35 |
| LTF | 4057 | lactotransferrin(LTF) | 36 |
| KDELR3 | 11015 | KDEL endoplasmic reticulum protein retention receptor 3(KDELR3) | 37 |
| METTL7B | 196410 | methyltransferase like 7B(METTL7B) | 38 |
| DPP4 | 1803 | dipeptidyl peptidase 4(DPP4) | 39 |
| APOBEC3F | 200316 | apolipoprotein B mRNA editing enzyme catalytic subunit 3F(APOBEC3F) | 40 |
| THBS1 | 7057 | thrombospondin 1(THBS1) | 41 |
| SOCS1 | 8651 | suppressor of cytokine signaling 1(SOCS1) | 42 |
| FCGR2C | 9103 | Fc fragment of IgG receptor IIc (gene/pseudogene)(FCGR2C) | 43 |
| PTPN22 | 26191 | protein tyrosine phosphatase, non-receptor type 22(PTPN22) | 44 |
| FCGR2B | 2213 | Fc fragment of IgG receptor IIb(FCGR2B) | 45 |
| MAPK13 | 5603 | mitogen-activated protein kinase 13(MAPK13) | 46 |
| GALNT5 | 11227 | polypeptide N-acetylgalactosaminyltransferase 5(GALNT5) | 47 |
| CARD16 | 114769 | caspase recruitment domain family member 16(CARD16) | 48 |
| TRIM6 | 117854 | tripartite motif containing 6(TRIM6) | 49 |
| SH2D2A | 9047 | SH2 domain containing 2A(SH2D2A) | 50 |
| CLCF1 | 23529 | cardiotrophin-like cytokine factor 1(CLCF1) | 51 |
| TMEM71 | 137835 | transmembrane protein 71(TMEM71) | 52 |
| NNMT | 4837 | nicotinamide N-methyltransferase(NNMT) | 53 |

**Plasmid descriptions**

| **Plasmid** | **Description** | **Source** |
| --- | --- | --- |
| pcDNA3.1A(+)-myc-His | hDDIT4L | this study |
| pcDNA3.1A(+)-myc-His | hDDIT4L Δ1 delete 96-106 | this study |
| pcDNA3.1A(+)-myc-His | hDDIT4L Δ2 delete 125-132 | this study |
| pcDNA3.1A(+)-myc-His | hDDIT4L Δ3 delete 141-166 | this study |
| pcDNA3.1A(+)-HA | hATP5B-HA | this study |
| pcDNA3.1A(+)-HA | hATP5A1-HA | this study |
| pcDNA3.1A(+)-HA | hATP5A1-HA Δ1 delete 118-131 | this study |
| pcDNA3.1A(+)-HA | hATP5A1-HA Δ2 delete 132-158 | this study |
| pcDNA3.1A(+)-HA | hATP5A1-HA Δ3 delete 179-192 | this study |
| pcDNA3.1A(+)-HA | hATP5A1-HA Δ4 delete 471-486 | this study |
| pcDNA3.1A(+)-HA | hATP5A1-HA Δ5 delete 491-504 | this study |
| pcDNA3.1A(+)-myc/His | hATP5A1-myc | this study |
| pcDNA3.1A(+)-myc/His | hATP5B-myc | this study |
| pGEX-4T-1 | GST-hATP5A1-HA | this study |
| pGEX-4T-1 | GST-hATP5B-HA | this study |
| pGEX-4T-1 | GST-hDDIT4L | this study |
| pcDNA3.1A(+)-HA | hTOM20-HA | this study |
| pcDNA3.1A(+)-HA | hTOM22-HA | this study |
| pcDNA3.1A(+)-HA | hTOM40-HA | this study |
